# Assessing inequality of opportunity in access to maternal healthcare services in pakistan: A quantitative attempt

**DOI:** 10.1186/s12913-025-13312-5

**Published:** 2025-09-01

**Authors:** Jawad Rahim Afridi, Sajjad Ahmad Jan, Muhammad Farhan Asif

**Affiliations:** 1 Department of Economics, University of Peshawar, Peshawar, Pakistan; 2 2UKM-Graduate School of Business, Universiti Kebangsaan Malaysia, Bangi, Selangor Malaysia; 3 Department of Business Management, Ilma University, Karachi, Pakistan

**Keywords:** MHCS, Inequality of opportunity, Pakistan, PDHS

## Abstract

**Background:**

Maternal healthcare services are an important goal in SDGs around the world. Persistent inequalities and gaps in maternal healthcare access are strongly linked to poor maternal health outcomes. This study aims to evaluate inequality of opportunity in access to maternal healthcare services by assessing the contributions of circumstances and efforts to these inequalities. Additionally, it analyzes the key determinants influencing the utilization of maternal healthcare services in Pakistan.

**Method:**

Using data from the Pakistan Demographic Health Surveys (2012–13 and 2017–18), the research examines determinants of MHCS access, focusing on circumstances and efforts and their contribution to IO. This study uses the human opportunity index, Shapley decomposition index, and logistic regression.

**Result:**

The Human Opportunity Index findings indicate notable improvements in antenatal care (from 25.70% in 2013 to 37.78% in 2018) and skilled birth attendance (from 38.97 to 55.73%), while postnatal care coverage declined significantly (from 35.56 to 22.56%) over the same period, underscoring the need for increased policy attention to postnatal services. Decomposition analysis reveals that the main circumstantial factors contributing to inequality are place of residence, region, and household wealth, while factors like the husband’s education and proximity to healthcare facilities play a minor role. Efforts such as women’s education, exposure to mass media, and women’s autonomy in health decisions have a significant impact on the inequality in MHCS access. Binary logistic regression analysis indicates that the probability of accessing MHCS varies by region, with women in Sindh having higher odds compared to those in Khyber Pakhtunkhwa (KP) and Balochistan. Urban women are more likely to receive skilled birth attendance and postnatal care than rural women. Women with basic education and decision-making power regarding their health are more likely to use MHCS. The study emphasizes the need for targeted interventions to address these disparities and improve maternal healthcare outcomes in Pakistan.

## Introduction

Economists have increasingly recognized that inequality of opportunity (IO), rather than income disparities alone, poses a major barrier to development in low- and middle-income countries. IO arises when circumstances beyond an individual’s control—such as gender, ethnicity, caste, or birthplace—limit access to essential services like education, healthcare, and employment [28]. Among these, healthcare is both a basic human right and a key driver of economic growth. Poor health restricts employment and income-generating opportunities, while good health enhances productivity and overall well-being. Equitable access to maternal healthcare services (MHCS) is especially crucial, as women from marginalized groups often face the greatest barriers to care. These disparities reinforce broader patterns of social and economic inequality. As emphasized in the Sustainable Development Goals (SDGs), universal access to healthcare is vital for reducing inequality and promoting sustainable development. However, despite global progress, significant gaps in maternal healthcare access persist in low-income countries [46].

Despite international initiatives to enhance maternal health, Pakistan still faces significant obstacles. According to the [47] the nation continues to struggle to provide safe delivery and adequate maternal care, with a maternal mortality rate of 186 deaths per 100,000 live births. These results highlight systemic disparities in healthcare, especially in rural areas. Basic health services are absent in about 30% of rural areas, and many of the clinics that do exist have ongoing staffing and medication shortages, with only 0.7 doctors for every 1,000 residents. Furthermore, timely access to care is hampered by the fact that more than 40% of rural women must travel more than 30 km to reach a healthcare center. These barriers reflect broader patterns of inequality of opportunity and reinforce the urgent need for inclusive health policies. Reducing avoidable maternal deaths and improving health outcomes in Pakistan depends on closing these service gaps and ensuring equitable access to care.

Both personal efforts and outside circumstances contribute to the inequalities in maternal health care access [34]. Household circumstances, the husband’s educational background, regional differences, and financial standing are examples of external variables. Individual activities, meanwhile, include mass media exposure, women’s education, work, and personal health decisions. Together, these elements influence the standard and availability of maternal healthcare services, illuminating the intricate relationship between individual preferences and structural impediments.

A diversified strategy is required to lessen maternal healthcare disparities in Pakistan. Important actions include bolstering the healthcare system, expanding access to healthcare in remote areas, and encouraging women’s economic and educational empowerment. Maternal health opportunities can be improved by addressing both individual and systemic barriers, which will lessen inequality in Pakistan and promote wider socioeconomic growth.

Maternal healthcare usage in Pakistan reflects a concerning trend. This study aims to evaluate inequality of opportunity in access to maternal healthcare services by assessing the contributions of circumstances and efforts to these inequalities. Additionally, it analyzes the key determinants influencing the utilization of maternal healthcare services across Pakistan.

### Literature review

Inequality of Opportunity (IO) has become a crucial lens for understanding persistent differences in health, education, and economic outcomes among nations. In contrast to traditional income inequality, IO focuses on how circumstances that is out of an individual’s control, like gender, ethnicity, location of birth, parental education, and socioeconomic background, limit their access to opportunities and necessary services. According to Marrero and Rodriguez [24], this paradigm identifies structural hurdles that impede social mobility and economic advancement by perpetuating inequality across generations [24].

Much of the early study on inequality of opportunity (IO) centered on Brazil, European Union countries, and the United States. For example, [24] examined how macroeconomic factors affect IO and inequality of effort (IE) using data from the United States Panel Study of Income Dynamics from 1970 to 2009. They discovered that higher real GDP and increasing consumer debt were related to lower IO, whereas welfare spending had a negative effect on IO and inflation positively influenced IE. These findings highlight the complex interplay between economic policy, macroeconomic conditions, and unequal consequences.

Globally, IO research repeatedly shows a substantial link between income inequality and opportunity gaps, with family background, parental education, and geographic location being important factors [33, 39]. [38] for example, found noteworthy regional disparities in healthcare access throughout India, demonstrating that areas with less developed infrastructure and less educated parents have less access to healthcare and basic nutrition. These revelations highlight the reality that inequality has multiple dimensions and is influenced by social and economic variables.

### Socioeconomic determinants of maternal healthcare in Pakistan and South Asia

Socioeconomic determinants have a significant impact on maternal healthcare consumption in South Asia, particularly Pakistan, according to the study, which supports wider IO patterns. According to [6] cultural prejudices that impede the development of female human capital are the reason behind Pakistan’s gender gaps in educational opportunities, where female net enrollment (54%) is behind males (61.1%). Female pupils do worse in reading and math, reflecting this disparity in academic attainment. It is advised that policy solutions focus on improving women’s access to financial resources and economic engagement in order to lessen these disparities in schooling.

To investigate IO in Pakistan’s labor market and household incomes, [35] employed both parametric and non-parametric methodologies. Their examination of the Pakistan Social and Living Standard Measurement Surveys (2005-06, 2010-11) revealed a significant 16% drop in family income IO and an 11-point drop in salary inequality, suggesting some progress but also enduring differences related to parental education, employment, gender, and location.

In Pakistan, socioeconomic issues also limit access to maternal healthcare. Maternal education, family affluence, media exposure, and women’s autonomy are identified as significant determinants impacting the use of prenatal, delivery, and postnatal care [3, 21, 49]. For example, [21] observed a 12% rise in maternal healthcare use between 2006 and 2012, owing mostly to changes in these variables. However, rural women confront significant hurdles, including limited access to healthcare services and traditional social norms that constrain autonomy, exacerbating inequality.

In their qualitative analysis of Punjabi maternal healthcare hurdles, [26] found that the main challenges faced by underprivileged women were social reliance and financial limitations. Their findings highlight the significance of focused policies addressing exclusion and inequality by demonstrating how national and local health programs frequently fall short in reaching disadvantaged people.

### Comparative evidence from other developing countries

Comparative studies conducted in other low- and middle-income nations support similar trends. To determine the main determinants influencing the use of antenatal care (ANC) in Cameroon, [48] used multilevel modeling and geographical analysis of data from the Demographic and Health Survey. Significant differences between urban and rural regions were discovered, and a large portion of the inequality was explained by factors such as household wealth, education, and place of residence. They supported recommendations for policies aimed at removing these hurdles by using Shapley and Fields’ decomposition techniques to highlight the part that changeable socioeconomic constraints play in restricting ANC access.

Similar to this,[43] examined data from the Demographic Health Survey conducted in Ethiopia in 2011, 2016, and 2019 and found that ANC coverage increased gradually from around 30–44.7%. Inequities still exist, though, and they disproportionately benefit wealthier women. ANC usage is highly predicted by factors such as mother’s age, education, geography, and experience of domestic abuse; financial level accounts for more than two-thirds of the disparities that have been reported. Their findings highlight how urgent it is to increase access to healthcare and enhance its quality for women from low-income backgrounds.

Alam et al. [5] examined the inequalities in maternal healthcare usage between urban and rural areas of Bangladesh. They found that the likelihood of utilizing trained birth attendants, facility-based delivery, and prenatal care is significantly increased by greater family wealth and partner and mother education levels. According to the report, socioeconomic inequality is a major factor in the ongoing discrepancies between urban and rural areas, and addressing these issues is crucial to achieving the Sustainable Development Goals (SDGs).

### Health inequality and child well-being: broader opportunity gaps

Research on inequality of opportunity also includes child well-being in addition to maternal healthcare. Using data from the Multiple Indicator Cluster Survey (MICS), [4] used the Human Opportunity Index (HOI) and Shapley decomposition to assess opportunity disparity in Pakistani children’s schooling, nutrition, and cognitive abilities. According to their findings, the main causes of differences in eating, reading, numeracy, and education were household economic status and place of residence, highlighting the critical influence of socioeconomic environment on early-life possibilities.

Similarly, in 39 and 41 nations, respectively, [17] discovered a positive correlation between income inequality and opportunity disparity. The Human Development Index (HDI) and IO were correlated in their research, which demonstrated that bigger income inequalities and decreased intergenerational mobility are associated with more opportunity inequality.

These results demonstrate how economic inequality, health, and education are intertwined and how crucial it is to use integrated strategies to address these overlapping aspects of inequality of opportunity.

### Geographic and policy implications: the case of Pakistan

In Pakistan, maternal health inequities are made worse by geographical and infrastructure differences. Balochistan, one of the least developed provinces, has the greatest death clusters, according to [25] geographical analysis of district-level maternal and newborn mortality. Their results indicate that specific expenditures in health infrastructure and public awareness are crucial since they demonstrate that wealth, sanitation, hand washing, and prenatal care coverage greatly lower mortality.

Furthermore, the lack of basic health facilities in 30% of rural regions, the doctor-patient ratio of 0.7 per 1,000 people, and the high travel distances (over 30 km for 40% of rural women) pose significant obstacles to equitable treatment (WHO, 2023). Policy changes are urgently needed to improve healthcare infrastructure, enhance service quality, and empower marginalized women because of these structural disparities and socioeconomic marginalization [31].

The need for more inclusive and locally customized programs is highlighted by national studies that also show that social, cultural, and economic exclusion endures in spite of governmental initiatives [26, 35]. Work in Nigeria that emphasizes culturally responsive messaging to reduce health inequalities has demonstrated the effectiveness of strategic communication and community participation in increasing awareness and building trust [15].

Inequality of opportunity remains a significant barrier to equitable maternal healthcare and broader socioeconomic progress in Pakistan and other developing countries. Research consistently shows that access to maternal health services is shaped by factors such as socioeconomic status, parental education, gender, location, and cultural norms. Addressing these challenges requires more than just financial assistance—comprehensive, targeted policies are needed to overcome economic, geographic, and educational barriers and ensure fair access to healthcare for marginalized groups.

### Methodology and data source

The Human Opportunity Index (HOI), as established by [13] and [2] assesses inequality of opportunity by evaluating both opportunity coverage and equitable distribution. It assesses the equitable distribution of opportunities such as education and healthcare across society on a scale of 0 to 100. The range of HOI is 0 to 100; 0 means no opportunity, while 100 means equal opportunity. [40] defined HOI as “the extent of a society’s current opportunities that are accessible and have been allocated fairly.”

.The Human Opportunity Index (HOI) is calculated as:

$$\:\text{H}\text{O}\text{I}=\stackrel{-}{\text{p}}\left(1-\text{D}\right)\:$$

Where:

$\:\stackrel{-}{\text{p}}$ is the average coverage rate, and.

$\:\left(1-\text{D}\right)\:$is the dissimilarity index (D-index).

The term (1 - D) represents the fraction of available opportunities that are evenly distributed. Thus, HOI reflects the average service coverage rate, adjusted by the dissimilarity index. When services are distributed equally, D = 0, meaning HOI equals the average coverage rate, indicating perfect equality of opportunity. Conversely, if no one in society can access services, D = 1, resulting in an HOI of zero, signifying no access to basic services.

The formula for average coverage is:

$$\:\stackrel{-}{\text{p}}={\sum\:}_{\text{i}=1}^{\text{n}}{\text{w}}_{\text{i}}{\text{p}}_{\text{i}}$$

Where:

w_i_ =1/n here “n” means the sampling weights.

$\:\stackrel{-}{\text{p}}$i is the overall coverage rate, and.

$\:{\text{p}}_{\text{i}}\:$is the coverage rate for each group based on circumstances or effort.

The D-index measures the disparity in access to services among groups with the same circumstances or effort. It calculates the proportion of opportunity that must be reallocated to achieve equality of opportunity. The formula for the D-index is:

$$\:\text{D}-\text{i}\text{n}\text{d}\text{e}\text{x}=\frac{1}{2\stackrel{-}{\text{p}}}{\sum\:}_{\text{i}=1}^{\text{n}}{\text{w}}_{\text{i}}\left|{\text{p}}_{\text{i}}-\:\stackrel{-}{\text{p}}\right|$$

The D-index ranges from 0 to 1, where 0 indicates perfect equality and 1 represents perfect inequality. As the D-index value increases, inequality rises. Although HOI reflects the overall level of opportunity (in)equality, it does not reveal the underlying factors, such as circumstances and effort, driving this inequality. To understand the contribution of these factors, the Shapley Decomposition Index is used.

### Shapley value for decomposition of the dissimilarity index

According to the Human Opportunity Index, the Shapley value decomposition, which is based on cooperative game theory [37], measures how much each variable contributes to inequality of opportunity. In this paper, shapley value decomposition is used to assess that how these factors i.e. circumstances and efforts marginally contribute in inequality of opportunity of maternal health care services in Pakistan. The World Bank [45], Aran & Ersoda, [7] and [2] all employ this approach to evaluate the variables affecting maternal healthcare services (MHCS) inequality.

Formula of Shapley value for Decomposition:

$$\:{\text{D}}_{{\text{p}}_{\text{j}}}={{\Sigma\:}}_{\text{S}\subseteq\:\text{N}/{\text{p}}_{\text{j}}}^{\frac{\left|\text{s}\right|!\left(\text{n}-\left|\text{s}\right|-1\right)!}{\text{n}}}[\text{D}\left(\text{S}\cup\:\left\{{\text{p}}_{\text{j}}\right\}\right)-\text{D}\left(\text{S}\right)]$$

Where.

N = Total number of circumstances or efforts selected for study i.e. women’s education or employment.

n = Total number of circumstances or efforts in set N.

S = Subset of circumstances or efforts that does not include p_j_.

D(S) = Function of subset of circumstances or efforts that does not include p_j_.

D(S ∪ {$\:{\text{p}}_{\text{j}}$}) = Function of subset of circumstances or efforts combined with p_j_.

The marginal contribution of each circumstances or efforts p_j_ to the dissimilarity index can be calculated as follows:

$$\:{{\uptheta\:}}_{{\text{p}}_{\text{j}}}=\frac{{\text{D}}_{{\text{p}}_{\text{j}}}}{\text{D}\left(\text{N}\right)},\:\:\:\:\:\:\:\:\:\:\:\:\text{w}\text{h}\text{e}\text{r}\text{e}\:{{\Sigma\:}}_{\text{i}\in\:\text{N}}{\text{D}}_{{\text{p}}_{\text{j}}}=1$$

​​​$\:{{\uptheta\:}}_{{\text{p}}_{\text{j}}}$, expresses the proportionate contribution of circumstance or effort P_j_ to the total value D(N) in a Shapley decomposition. The normalization condition $\:{{\Sigma\:}}_{\text{i}\in\:\text{N}}{\text{D}}_{{\text{p}}_{\text{j}}}=1$ ensures that the sum of the contributions from all circumstances or efforts P_j_ in the set N equals 1. This approach provides valuable insights into how various circumstances or efforts influence overall inequality of opportunity in case of Pakistan.

We assess the Human Opportunity Index (HOI) and apply the Shapley decomposition approach using statistical software i.e. STATA 15.1 version, to measure inequality of opportunity in access to maternal healthcare services in Pakistan.

### Econometric analysis

This study employs multivariable logistic regression to analyze the utilization of maternal healthcare services, including antenatal care (ANC), skilled birth attendance (SBA), and postnatal care (PNC). Each dependent variable is binary (e.g., whether a mother received a specific service), making logistic regression a suitable method for examining categorical outcomes. Multivariable logistic regression enables the assessment of the relationship between each binary outcome and multiple independent (predictor) variables, offering a robust framework for understanding the factors influencing maternal healthcare utilization.

The functional forms of model are given as below;

1 $$\begin{array}{c}\mathrm{MHCS}\;=\;\mathrm f\;(\mathrm{Circumstances},\;\mathrm{Efforts})\\\mathrm{MHCS}=\;\mathrm f\;(\mathrm{Residence},\;\mathrm{Region},\;\mathrm H.\mathrm{Edu},\;\mathrm W.\mathrm{Status},\;\mathrm{DNHF},\;\mathrm W.\mathrm{Edu},\;\mathrm W.\mathrm{Emp},\;\mathrm{EMM},\;\mathrm{WODH})\end{array}$$

2 $$\begin{aligned} \:\left(p\right)=&\frac{1}{1-p}=MHCS={\beta\:}_{0}+{\beta\:}_{1}\left(RESIDENCE\right)\\&+\:{\beta\:}_{2\:}\left(REGION\right)+\:{\beta\:}_{3\:}\left(H.EDU\right)\\&+{\beta\:}_{4\:}\left(W.STATUS\right)+\:{\beta\:}_{5\:}\left(DNHF\right)\\&+\:{\beta\:}_{6\:}\left(W.Edu\right)+{\beta\:}_{7\:}\left(W.EMP\right)\\&+\:{\beta\:}_{8\:}\left(EMM\right)+\:{\beta\:}_{9\:}\left(WODH\right)+\:\mu\:\: \end{aligned}$$

The probability of the feature of interest being present is denoted by p. The logged odds are used to define the Logit transformation:

$$\:Odds=\:\frac{p}{1-p}$$

or

$$\:logit\left(p\right)=\text{l}\text{n}\left(\frac{p}{1-p}\right)$$.

Maternal health care services (MHCS), as defined by the WHO, include care during pregnancy, childbirth, and the postpartum or postnatal period. The study examines three key service variables: Antenatal Care (ANC), Skilled Birth Attendance (SBA), and Postnatal Care (PNC). ANC is recorded as 1 for women with at least four antenatal visits, and 0 otherwise. SBA is marked as 1 for competent birth attendance and 0 for non-competent attendance. PNC is coded as 1 if care is received within 42 days post-birth and 0 if not. These variables assess women’s access to essential maternal healthcare services.

This study investigates factors influencing maternal healthcare services, considering both circumstance and effort variables. Circumstance variables include area of residence (rural = 0, urban = 1), household wealth (ranked from 1 for the poorest to 5 for the richest), distance to health facilities (0 = No, 1 = Yes), and husband’s education level (ranging from 0 for no education to 3 for higher education). Effort variables include women’s education (0 for no education to 3 for higher education), employment status (0 = not working, 1 = working), media exposure (1 for exposure to family planning messages, 0 for no exposure), and participation in healthcare decision-making (1 for participation, 0 for non-participation).

### Data source

This study uses data from the Pakistan Demographic and Health Surveys (PDHS) 2012–13 and 2017–18[32]. Both surveys employed a two-stage sampling technique. PDHS 2012–13 sampled 14,000 households (6,944 urban, 7,056 rural), with 7942 respondents after removing missing data. PDHS 2017–18 selected 580 sampling units (295 rural, 285 urban), with 16,240 households (8,260 rural, 7,980 urban). A total of 50,495 married women were surveyed. We examined the household characteristics dataset, ensuring relevance to study indicators. After data analysis, 7,887 observations remained for analyzing inequality of opportunity in maternal healthcare services (MHCS) in Pakistan.

## Results and interpretations

### Human opportunity index (HOI)

The Human Opportunity Index (HOI) measures a person’s ability to accomplish a desired objective, such as access to education, healthcare, or an acceptable standard of living, independent of their socioeconomic status. It is a composite indicator that includes assessments of access, coverage, and quality of essential services, with a focus on the distribution of opportunities across socioeconomic groups.

The Human Opportunity Index (HOI) was developed by World Bank economists to evaluate the extent to which a country provides its citizens with equal opportunities. The index measures the extent to which residents, regardless of socioeconomic class, has access to basic services and opportunities including education, healthcare, and clean water. Table 1 provides inequality of opportunity results based on human opportunity index of Pakistan as follows;

**Table 1:** Distribution of the coverage, dissimilarity index, and HOI of Pakistan

Health Care Service	Average Coverage Rate or Prevalence %		Inequality of opportunity D%		(1-D)%		Human Opportunity Index %	
	2013	2018	2013	2018	2013	2018	2013	2018
Antenatal Care	34.40	48.97	25.40	22.80	74.60	77.20	25.70	37.78
Skilled Birth Attendance	49.14	64.68	20.69	13.82	79.31	86.18	38.97	55.73
Postnatal Care	43.12	26.72	17.63	17.56	82.37	82.44	35.52	22.56

### Human opportunity index of antenatal care service of Pakistan

Table 1 shows considerable increases in antenatal care (ANC) service coverage in Pakistan from 2013 to 2018. The coverage percentage rose from 34.40% in 2013 to 48.97% in 2018, indicating an improvement in maternal healthcare availability. The Human Opportunity Index (HOI) for ANC services increased from 25.7 to 37.78%, showing that more women had access to critical maternity healthcare treatments. At the same time, the Dissimilarity Index (D-index) fell from 25.40 to 22.80%, indicating a decrease in inequality of opportunity in accessing ANC.

This upward tendency can be attributed to a variety of socioeconomic reasons. Female literacy increased from 49 to 64% within the same time period, which had an important influence on enhancing maternal healthcare usage. Education impacts women’s healthcare decisions because those with higher education levels are more likely to seek timely and regular ANC visits. Women with elementary and secondary education had greater ANC usage rates than illiterate women, but highly educated women start care earlier and attend more appointments. This is consistent with research from Nepal and Ethiopia, which both stress the impact of education on maternal healthcare access.

Access to ANC has improved as a result of urbanization. People who relocate from rural to urban regions have easier access to medical treatment. Due to a lack of proper healthcare facilities, rural women—who frequently have lower educational attainment and greater unemployment rates—had difficulty accessing ANC. On the other hand, metropolitan women use ANC more frequently because they have more access to economic and educational prospects. This urban advantage supports the need for focused measures to close the healthcare gap between rural and urban areas and is consistent with findings from previous research.

Maternal healthcare access has also been improved by increased healthcare facilities, universal health coverage, media awareness campaigns, better sanitation, and different family wealth levels. Table 1’s trends demonstrate that although Pakistan has made strides, more policy work is required to maintain gains and further lower inequality.

### Human opportunity index of skilled birth attendance service of Pakistan

Table 1 demonstrates that SBA coverage in Pakistan grew from 49.14% in 2013 to 64.68% in 2018, showing more access to trained birth attendants. The Dissimilarity Index (D-index) for SBA decreased from 20.69 to 13.82%, indicating less inequality in access. The proportion of newborns attended by competent professionals increased from 79.31 to 86.18%, while SBA’s Human Opportunity Index (HOI) increased from 38.97 to 55.73% in five years.

Several reasons contribute to this development, including urbanization, increasing female education, enhanced healthcare facilities, and increased media awareness. Rural women have limited educational and healthcare options, generally depending on traditional birth attendants. Higher levels of female education, on the other hand, contribute to increased healthcare knowledge and usage because educated women make better decisions and have more confidence in obtaining expert delivery care.

Employment also impacts SBA usage, with working women more likely to choose public healthcare due to financial incentives. Increased government healthcare funding, nurse and midwife recruitment, and infrastructural expansion all improve SBA access. Despite these advances, Pakistan’s SBA access remains below the worldwide average, demanding more policy interventions to close the gap.

### Human opportunity index of postnatal care service of Pakistan

Pakistan’s postnatal care coverage decreased from 43.12% in 2013 to 26.72% in 2018, as shown in Table 1. Persistent inequality was reflected in the Dissimilarity Index (D-index), which stayed relatively constant, dropping somewhat from 17.63 to 17.56%. The Human Opportunity Index (HOI) similarly showed decreased accessibility, falling from 35.52 to 22.56%.

Education, family structure, work, household affluence, media exposure, and male engagement are some of the factors that affect the usage of postnatal care. Women in extended families and those living in rural areas face more obstacles. Socioeconomic differences still exist, but access is better for wealthier households and women with more education. Given that Pakistan’s postnatal care coverage is still below the worldwide average, closing educational inequalities, boosting healthcare in rural areas, and encouraging women’s empowerment are crucial to improving maternal health.

### Change in the human opportunity index of MHCS

**Fig. 1:**
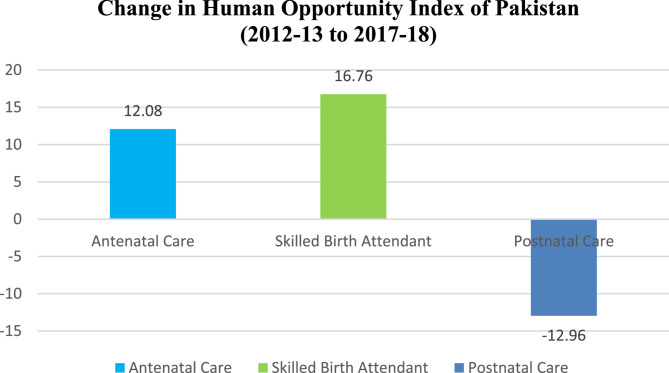
Change in the Human Opportunity Index of Pakistan (2012-13 to 2017-18)

Women who delivered with a trained birth attendant had the greatest human opportunity index score, whereas postnatal care had the lowest. A low human opportunity index score indicates that women do not seek postnatal care within 40 days after giving birth. Human Opportunity Index (HOI) of Pakistan 29−18) change has been seen in Fig. 1.

### Variations in HOI for maternal healthcare (2012-13 to 2017-18)

In the case of Pakistan, Fig. 1 shows notable changes in the Human Opportunity Index (HOI) between 2012 and 13 and 2017-18 across key maternal healthcare services. Antenatal care and skilled birth attendance experienced improvements in equitable access, with HOI increases of 12.08% and 16.76%, respectively. In contrast, postnatal care saw a significant decline of 12.96%, indicating growing inequality in access.

Since the HOI accounts for coverage and how equitably opportunities are distributed, improvements in antenatal and delivery care suggest progress in reaching wider populations. However, the decline in postnatal care highlights persisting disparities—particularly affecting women in rural areas and those with lower education levels. The Dissimilarity Index (D-index), which reflects the inequality component of opportunity, indirectly contributes to these HOI trends. These findings underline the need for targeted policy interventions to address inequality in postnatal care and ensure balanced improvements across all maternal health services, especially for underserved communities in Pakistan.

### Shapley value decomposition of Pakistan’s inequality of opportunity

We use the Shapley decomposition approach to assess the marginal contribution of different circumstances and efforts to disparity in maternal healthcare access, as measured by HOI. The results show that household wealth, location, urban-rural residence, and husband’s degree are the most important factors influencing inequality of opportunity (IO) in maternal healthcare services. Table 2 quantifies the influence of these characteristics, which can help policymakers address key discrepancies.

**Table 2:** Shapley value decomposition of D-Index– percentage contributions of circumstances or efforts of MHCS in Pakistan

Circumstances/Efforts	Antenatal Care (ANC)		Skilled Birth Attendance (SBA)		Postnatal Care (PNC)	
	2013	2018	2013	2018	2013	2018
Region of Residence	15.10%	10.26%	17.11%	12.35%	26.16%	12.84%
Place of Residence	12.0%	9.37%	11.55%	9.72%	5.21%	5.35%
Husband Education	8.90%	11.51%	1.16%	10.71%	0.13%	5.22%
Women’s Education	13.0%	23.02%	27.49%	20.59%	26.02%	17.79%
Household Wealth Status	23.40%	23.92%	26.09%	25.57%	29.53%	12.77%
Distance to Nearest Health Facility	9.60%	8.95%	1.45%	10.12%	2.79%	10.62
Women Employment Status	1.2%	0.12%	2.26%	0.30%	0.84%	3.79%
Exposure to Mass Media	12.90%	8.54%	11.68%	6.94%	8.62%	7.88%
Women Own Decision about Health	3.90%	4.31%	1.16%	3.70%	0.64%	23.68%

### Maternal health care services in Pakistan: the marginal and relative contribution of circumstances and effort

Table 2 presents the Shapley value decomposition of the D-Index, a composite index measuring the quality of maternal health care services in Pakistan. ANC, SBA, and PNC are the three components of the D-Index.

The Shapley value decomposition technique is used to distribute the overall value of the D-Index to the various circumstances or efforts that contribute to it. The percentages in the table represent the proportional contribution of each circumstance and effort to the overall D-index, or inequality of opportunity value. The following presents the marginal and relative contribution of each circumstance and effort variable:

Region of Residence: Access to maternity healthcare is greatly impacted by geographic inequities. Residence region was responsible for 15.10% (ANC), 17.11% (SBA), and 26.16% (PNC) of inequality in 2013. These percentages fell to 10.26% (ANC), 12.35% (SBA), and 12.84% (PNC) by 2018. Balochistan and FATA have serious problems with healthcare access, but Punjab has greater access because of infrastructure and knowledge.

Place of Residence: Although they are decreasing, urban-rural discrepancies still exist. In 2013, 12% (ANC), 11.55% (SBA), and 5.21% (PNC) of inequality was caused by living in a rural area. These percentages dropped to 9.37% (ANC), 9.72% (SBA), and 5.35% (PNC) by 2018, indicating better rural healthcare services but still notable obstacles.

Husband Education: Education of husbands has a greater influence on ANC than SBA or PNC. In 2013, its contribution to inequality was 8.90% (ANC), 1.16% (SBA), and 0.13% (PNC), which increased to 11.51% (ANC), 10.71% (SBA), and 5.22% (PNC) by 2018. Husbands who are educated make maternal healthcare more accessible by raising awareness, providing financial support, and assisting with transportation.

Women’s education: A significant predictor of maternal healthcare consumption. In 2013, women’s education contributed 13% (ANC), 27.49% (SBA), and 26.02% (PNC) to income disparity. By 2018, its influence on ANC had grown to 23.02%, while it had decreased to 20.59% for SBA and 17.79% for PNC. Educated women are more likely to seek antenatal and professional delivery care.

Healthcare disparity is driven by disparities in household wealth. In 2013, wealth contributed 23.40% (ANC), 26.09% (SBA), and 29.53% (PNC) to income inequality. By 2018, its influence remained strong for ANC (23.92%) and SBA (25.57%), but declined for PNC (12.77%), showing that postnatal healthcare availability had improved.

Distance to health facilities is a significant challenge, especially for ANC patients. In 2013, it contributed 9.6% (ANC) to inequality, which fell somewhat to 8.95% (ANC), 10.12% (SBA), and 10.62% (PNC) in 2018. Rural women have additional hurdles due to inadequate infrastructure and expensive transportation costs.

Women’s Employment: Employment plays a minimal role in healthcare disparities. Its contribution was minimal in 2013, but it increased to 0.12% (ANC), 0.3% (SBA), and 3.79% (PNC) by 2018. Economic independence enhances healthcare options, particularly postnatal care.

Exposure to Mass Media: Media exposure reduces healthcare disparities. In 2013, it contributed 12.9% (ANC), 11.68% (SBA), and 8.62% (PNC) to inequality. By 2018, its influence declined to 8.54% (ANC), 6.94% (SBA), and 7.88% (PNC), reflecting increased awareness but a reduced impact on service access.

Women’s Decision-Making: Autonomy in health decisions is crucial for access. In 2013, its contribution was 3.9% (ANC), 1.16% (SBA), and 0.6% (PNC). By 2018, its impact increased to 4.31% (ANC), 3.7% (SBA), and 23.68% (PNC), emphasizing the need for greater empowerment in postnatal care decisions.

Maternal healthcare inequality in Pakistan is shaped by both circumstantial factors (region, wealth, education) and effort-based factors (employment, media exposure, autonomy). Over time, education and media have helped reduce disparities, especially in ANC and SBA. However, regional and wealth-based inequalities remain significant, particularly for PNC, necessitating targeted policy interventions.

### Association between socio-economic variables and maternal health care services

We present the relationship of region of residence, place of residence, women’s education, husband’s education, wealth status of women’s household, women’s decisions about their own health care, women’s employment status, exposure to mass media, and distance to the nearest health facility with MHCS, i.e., ANC, SBA, and PNC of Pakistan. Table 3 indicates the results of the relationship between socio-economic determinants and MHCS by using binary logistic regression models.

Maternal healthcare utilization in Pakistan is significantly influenced by region of residence, education levels, household wealth, media exposure, and healthcare accessibility. Disparities exist in antenatal care (ANC), skilled birth attendance (SBA), and postnatal care (PNC) across socioeconomic groups, necessitating targeted interventions to improve access.

### Region of residence

The region of residence has a substantial impact on maternal healthcare access. Table 3 from 2018 compares the odds ratios for Sindh, KP, Balochistan, ICT, GB, AJK, and FATA to Punjab. Sindh has better access than Punjab, with KP and Balochistan trailing behind. ICT has higher SBA but lower ANC and PNC probability, whereas GB has enhanced ANC but lower SBA. AJK has reduced SBA chances, but no significant ANC or PNC differences. FATA has curtailed SBA and PNC access. These discrepancies underscore the need for targeted interventions to improve maternal healthcare in deprived areas [9, 49].

### Place of residence

The place of living has a substantial impact on maternal healthcare access. Table 3 from 2018 reveals that rural women are less likely than urban women to receive ANC, SBA, or PNC. Healthcare is more readily available in urban places. Studies from Nigeria, India, Zimbabwe, Switzerland, Kenya, Ethiopia, and Pakistan confirm that maternal healthcare is more commonly used in cities [12, 16, 39, 49].

### Husband education

A husband’s education has a considerable impact on maternal healthcare use. Table 3 demonstrates that greater education levels are associated with increased maternal healthcare consumption, with the highest education group having the greatest impact. The influence varies by service; for ANC, basic education has a larger likelihood than secondary or higher education. Overall, a husband’s education has a significant impact on postnatal care utilization [22, 23].

### Women’s education

Women’s education has a major impact on maternal healthcare consumption. Table 3 demonstrates a clear positive link between education levels and access to ANC, SBA, and PNC, with “No Education” as the reference group. Even after controlling for other variables, more education considerably increases the likelihood of maternal healthcare utilization. Educated women are more knowledgeable about health issues, available options, and the importance of maternal care. These findings are consistent with previous research from Peru [14], the Philippines [10], Sub-Saharan Africa [18], and Pakistan [27].

**Table 3:** Determinants of Maternal Health Care Services in Pakistan (Binary Logistic Regression)

Independent Variables		Antenatal Care (2018) Binary Logistic Regression		Skill Birth Attendance Care (2018) Binary Logistic Regression		Postnatal Care (2018) Binary Logistic Regression	
		Beta$(\beta )$	Odds Ratio	Beta$(\beta )$	Odds Ratio	Beta$(\beta )$	Odds Ratio
Region of Residence	Punjab	Reference					
	Sindh	0.34***	1.41	0.75***	2.12	0.33***	1.40
	KP	-0.11	0.89	− 0.003	0.99	-0.69***	0.50
	Balochistan	-0.80***	0.44	-0.87***	0.41	-0.84***	0.42
	ICT	-0.02	0.97	0.65***	1.91	-0.75***	0.47
	GB	0.62***	1.87	-0.03	0.96	0.06	1.06
	AJK	-0.19**	0.82	-0.15**	0.85	0.03	1.03
	FATA	-0.24**	0.78	0.39***	1.479	-1.86***	0.15
Place of Residence	Urban	Reference					
	Rural	-0.18**	0.82	-0.26***	0.77	0.02**	0.83
Husband Education	No Education	Reference					
	Primary	0.18**	1.20	0.18***	1.20	-0.00	0.99
	Secondary	0.35***	1.43	0.17***	1.18	0.07	1.08
	Higher	0.38***	1.47	0.30***	1.35	0.018	1.019
Women’s Education	No Education	Reference					
	Primary	0.45***	1.57	0.31***	1.37	0.01***	1.01
	Secondary	0.70***	2.02	0.66***	1.94	0.03**	1.04
	Higher	1.34***	3.83	1.26***	3.55	0.38***	1.47
Decision About Own Health	No	Reference					
	Yes	0.14***	1.15	0.10***	1.11	0.29***	1.34
Wealth Status of Women’s Household	Poorest	Reference					
	Poorer	0.47***	1.60	0.53***	1.70	0.09	1.09
	Middle	0.83***	2.30	1.05***	2.85	0.11	1.12
	Richer	1.18***	3.25	1.38***	3.97	0.12	1.13
	Richest	1.87***	6.54	1.91***	6.78	0.38***	1.46
Women’s Employment Status	Unemployed	Reference					
	Employed	-0.07	0.93	-0.01	0.98	0.13*	1.14
Exposure to Mass Media	No	Reference					
	Yes	-0.009	0.99	0.15***	1.19	0.09	1.10
Distance From Nearest Health Facility	No	Reference					
	Yes	-0.14**	0.86	-0.19***	0.82	0.00	1.00
Nagelkerke R²(Goodness of Fit)		0.34.2 (34.2%)		0.286 (28.6%)		0.36.9 (36.9%)	

*p < 0.10, **p < 0.05, ***p< 0.01

### Women’s decision about own health

Table 3 use binary logistic regression to examine the link between a woman’s health choices and three dependent variables: ANC, SBA, and PNC. The independent variable is categorized into two options: “No” and “Yes.” Table 3 presents the findings of the 2018 study and reveals a favorable correlation between a woman’s health choice and receipt of all three health services (ANC, SBA, and PNC). It indicates that there is a larger favorable correlation between a woman’s health choice and all three health services [19, 42].

### Wealth status of women’s household

According to Table 3, people who are wealthier are more likely to use ANC, SBA, and PNC [44]. Compared to women in the lowest wealth category, middle-class women had better access to maternal healthcare. Financial protection, better health infrastructure, and health education should be the main priorities in order to lessen inequities [1].

### Women’s employment status

In 2018, employed women were more likely to use PNC than ANC or SBA, according to Table 3. Maternal healthcare utilization is impacted by employment, necessitating specific strategies. Nonetheless, expert birth services are more frequently used by unemployed mothers. Low pay and household responsibilities are obstacles [41].

### Exposure to mass media

In Pakistan, maternal healthcare utilization is greatly influenced by exposure to the mass media. Table 3 demonstrates a small impact on ANC and PNC but a high correlation between media exposure and SBA. Like formal education, media initiatives raise awareness of health issues. The relationship between maternal healthcare utilization and health knowledge has been confirmed by earlier studies conducted in Pakistan and Morocco [8, 20].

### Distance to nearest health facility

The distance between healthcare institutions has a substantial impact on maternal healthcare utilization. Table 3 reveals that ANC and SBA are directly related to proximity to health facilities, although PNC has no significant effect. Limited access to healthcare, particularly antenatal and delivery care, has a negative impact on mother and child health outcomes. Poor infrastructure, a lack of major highways, and distant living circumstances further limit access. The majority of healthcare facilities are located in urban regions, leaving rural communities neglected. Previous research in rural Mali, Haiti, Guatemala, Uruguay, Malawi, Sub-Saharan Africa, and Pakistan confirms that spatial constraints have a detrimental impact on maternal healthcare usage [11, 19, 27, 30, 36].

The results of Nagelkerke R² indicate that all three models of determinants of maternal healthcare services in Pakistan are a good fit; i.e., the ANC model explained 34.2% of variables, the SBA explained 28.6%, and the PNC model explained 36.9% of variables. Because in social sciences, health studies, and public policy research, these values are generally considered adequate or even strong fits, particularly in areas where human behavior and social factors influence the outcomes and where achieving high R² values (e.g., over 50%) can be difficult. The study emphasizes the impact of socio-economic factors on maternal healthcare utilization in Pakistan, covering antenatal, delivery, and postpartum care. Key determinants include regional disparities, education levels, household wealth, media exposure, and healthcare accessibility. Women in underserved areas require targeted policies to improve access. Strengthening women’s education, economic stability, and media awareness can significantly enhance maternal healthcare uptake. Addressing these disparities through strategic interventions is essential for improving maternal and newborn health outcomes across the country.

The study emphasizes the impact of socio-economic factors on maternal healthcare utilization in Pakistan, covering antenatal, delivery, and postpartum care. Key determinants include regional disparities, education levels, household wealth, media exposure, and healthcare accessibility. Women in underserved areas require targeted policies to improve access. Strengthening women’s education, economic stability, and media awareness can significantly enhance maternal healthcare uptake. Addressing these disparities through strategic interventions is essential for improving maternal and newborn health outcomes across the country.

## Conclusion

The government of Pakistan has made concerted efforts to expand access to maternal healthcare services (MHCS) as a strategy to reduce maternal mortality and foster economic growth. However, inequality in MHCS continues to contribute significantly to maternal and neonatal mortality rates. This study investigates inequality of opportunity in maternal healthcare by employing the Human Opportunity Index (HOI) and Shapley decomposition methods to assess the influence of both individual circumstances and efforts. Using data from the Pakistan Demographic and Health Surveys (PDHS) from 2012 to 13 to 2017-18, the analysis reveals inequalities in access to maternal care based on geography, socioeconomic status, and education.

Although access to skilled birth attendants has improved, substantial gaps remain in antenatal and postnatal care, particularly in underserved populations, which poses ongoing risks to maternal and newborn health. To address these inequalities, the study recommends strengthening rural healthcare infrastructure, expanding maternal health education programs for women, increasing investments in the training of skilled birth attendants, and implementing targeted financial assistance schemes for disadvantaged households.

For sustainable improvements in maternal health and broader national development, continuous policy review and adaptive strategies are essential. Furthermore, the study’s impact would be enhanced by directly linking each empirical finding to specific, actionable policy recommendations to guide decision-makers more effectively.

### Policy recommendation

To reduce inequality in maternal healthcare access in Pakistan, the study suggests improving women’s education to enhance awareness and decision-making, expanding rural healthcare infrastructure, and addressing wealth disparities through progressive taxation and social programs. Promoting gender equality, increasing women’s employment, and investing in the healthcare workforce are also essential. Awareness campaigns, monitoring systems, and the development of nursing colleges are recommended. Finally, adopting effective maternal healthcare policies from developed countries can further strengthen outcomes and ensure equitable access nationwide.

## Limitations of the study

Our study relies on cross-sectional data from PDHS 2012–13 and 2017–18, which may not capture recent policy, infrastructure, or socioeconomic changes. Future research should use updated datasets and longitudinal studies to track trends and assess disparities over time. A panel data approach could strengthen causal inferences by addressing unobserved heterogeneity. Additionally, our focus on public healthcare excludes the private sector, NGOs, and informal providers, limiting the analysis of healthcare access disparities. Future studies should compare public and private services to assess cost, quality, and availability influences.A broader scope, including family planning, child healthcare, and family systems, would offer more profound insights into women’s healthcare needs. Regional disparities, especially in Balochistan and former FATA, should be analyzed for targeted policy recommendations. Expanding research in these areas will help reduce healthcare inequalities and promote equitable access for all women in Pakistan.

## Data Availability

The research paper is based on two data sets of Pakistan Demographic Health Survey (PDHS) 2012-13 & 2017-18.https://datacatalog.ihsn.org/catalog/4075https://datacatalog.ihsn.org/catalog/7970.

## References

[1] Adedokun ST, Uthman OA, Adekanmbi VT, Wiysonge CS. Incomplete childhood immunization in Nigeria: a multilevel analysis of individual and contextual factors. BMC Public Health. 2017;17(1):236. 28270125 10.1186/s12889-017-4137-7 PMC5341359

[2] Afridi JR, Jan SA. Assessment of inequality of opportunity in access to maternal healthcare services in Khyber pakhtunkhwa, Pakistan. Bull Bus Econ (BBE). 2024;13(2):870–6.

[3] Ahmad N, Hyder AA. Factors affecting prenatal care use in pakistan: A logistic regression analysis. East Mediterr Health J. 2016;22(4):262–9.

[4] Akram S, Pervaiz Z. Assessing Inequality of Opportunities for Child Well-being in Pakistan. Child Indic Res. 2025;18(2):525-42. 10.1007/s12187-024-10205-7.

[5] Alam AS, Alam S, Mobasshira K, Anik SN, Hasan MN, Chowdhury MAB, Uddin MJ. Exploring urban-rural inequalities of maternal healthcare utilization in Bangladesh. Heliyon. 2025;11(2):1–15. 10.1016/j.heliyon.2025.e41945 PMC11795031 39911430

[6] Aslam M. Education gender gaps in pakistan: is the labor market to blame? Econ Dev Cult Change. 2009;57(4):747–84.

[7] Aran MA, Ersado L. (2013). Inequality of opportunity in access to basic services among Egyptian children. Dev Analytics Res Paper Ser, *1304*.

[8] Aslam M, Kingdon GG. Parental education and child health—understanding the pathways of impact in Pakistan. World Development. 2012;40(10), 2014-2032.

[9] Babalola S, Fatusi A. Determinants of use of maternal health services in Nigeria-looking beyond individual and household factors. BMC Pregnancy Childbirth. 2009;9(1):43. 19754941 10.1186/1471-2393-9-43 PMC2754433

[10] Becker GS. A treatise on the family: Enlarged edition. Harvard University Press; 1993.

[11] Chidiebere ODI, Uchenna E, Kenechi OS. Maternal sociodemographic factors that influence full child immunization uptake in Nigeria. South African Journal of Child Health. 2014; 8(4), 138-142.

[12] Dar S, Afzal U. Education and maternal health in pakistan. The pathways of influence. Lahore J Econ. 2015;20(2):1–34.

[13] De Barros RP, Ferreira F, Vega J, Chanduvi J. Measuring inequality of opportunities in Latin America and the Caribbean. World Bank; 2009.

[14] Elo IT. Utilization of maternal health-care services in peru: the role of women’s education. Health Transition Rev. 1992;2(1):49–69. 10148665

[15] Ezeaka NB, Ochuba CC, Bartholomew CE. Addressing healthcare inequalities in nigeria: A communication perspective on advocacy and policy implications. J Adv Res Multidisciplinary Stud. 2025;5(1):1–11.

[16] Ezeonwu MC. Maternal birth outcomes: processes and challenges in Anambra state, Nigeria. Health Care Women Int. 2011;32(6):492–514. 21547803 10.1080/07399332.2011.555827

[17] Ferreira FH, Gignoux J. The measurement of inequality of opportunity: Theory and an application to Latin America. Review of Income and Wealth. 2011;57(4), 622-657.

[18] Fosu GB. Childhood morbidity and health services utilization: cross-national comparisons of user-related factors from DHS data. Soc Sci Med. 1994;38(9):1209–20. 8016686 10.1016/0277-9536(94)90186-4

[19] Gage AJ. Barriers to the utilization of maternal health care in rural Mali. Soc Sci Med. 2007;65(8):1666–82. 17643685 10.1016/j.socscimed.2007.06.001

[20] Glewwe P. Why does mother's schooling raise child health in developing countries? Evidence from Morocco. Journal of Human Resources. 1999;34(1), 124-159.

[21] Iqbal S, Maqsood S, Zakar R, Zakar MZ, Fischer F. Continuum of care in maternal, newborn and child health in Pakistan: analysis of trends and determinants from 2006 to 2012. BMC Health Serv Res. 2017;17(1):189. 28279186 10.1186/s12913-017-2111-9 PMC5345258

[22] Khan MA, Zahidie A. Impact of husband’s education on maternal healthcare utilization in Pakistan. J Ayub Med Coll Abbottabad. 2015;27(3):572–6.

[23] Khan N, Ahmed S, Khalique N, Siddiqui N, Kadir MM. Role of husband’s education in the utilization of maternal healthcare services in Pakistan. J Ayub Med Coll Abbottabad. 2019;31(4):561–5.

[24] Marrero GA, Rodríguez JG. Inequality of opportunity in Europe. Rev Income Wealth. 2012;58(4):597–621.

[25] Muhammad FS, Shahabudin SM, Talib MBA. Measuring spatial inequalities in maternal and child mortalities in Pakistan: evidence from geographically weighted regression. BMC Public Health. 2024;24(1):2229. 39152373 10.1186/s12889-024-19682-5 PMC11328511

[26] Mumtaz Z, Salway S, Bhatti A, Shanner L, Zaman S, Laing L, Ellison GT. Improving maternal health in pakistan: toward a deeper Understanding of the social determinants of poor women’s access to maternal health services. Am J Public Health. 2014;104(S1):S17–24. 24354817 10.2105/AJPH.2013.301377 PMC4011098

[27] Murtaza F, Mustafa T, Awan R. Determinants of no immunization of children under 5 years of age in Pakistan. J Family Community Med. 2016;23(1):32. 26929727 10.4103/2230-8229.172231 PMC4745199

[28] Nizamani S, Waheed MS. Do Pakistani Kids Have An Equal Chance? A Study of Inequality of Human Opportunities for Kids in Pakistan; 2020.

[29] Pakistan NIPS. (2013). Pakistan Demographic and Health Survey 2012-13 Islamabad. *Pakistan: National Institute of Population Studies and Macro International Inc.*

[30] Pebley AR, Goldman N, Rodriguez G. Prenatal and delivery care and childhood immunization in Guatemala: Do family and community matter? Demography. 1996; 33(2), 231-247. 8827167

[31] Pervaiz Z, Akram S. Estimating inequality of opportunities in Punjab (Pakistan): A Non-Parametric approach. Pakistan J Commer Social Sci. 2018;12(1):136–52.

[32] PDHS. (2018). Pakistan Demographic and Health Survey 2017-18. Available at: http://nips.org.pk/abstract_files/PDHS%202017-18%20-%20key%20%20findings.pdf

[33] Pistolesi N. Inequality of opportunity in the land of opportunities, 1968–2001. J Economic Inequal. 2009;7(4):411–33.

[34] Roemer, J. E. A pragmatic theory of responsibility for the egalitarian planner. Philos Public Affairs. 1993;22(2):146–66. https://www.jstor.org/stable/2265444.

[35] Shaheen S, Awan MS, Cheema AR. Measuring inequality of opportunity in Pakistan. Pak Econ Soc Rev. 2016;54(2):165–90.

[36] Shaikh S, Taj TM, Kazi A, Ahmed J, Fatmi Z. Coverage and predictors of vaccination among children of 1-4 years of age in a rural sub-district of Sindh. J Coll Physicians Surg Pak. 2010;20(12):806–10. 21205546

[37] Shorrocks AF. Decomposition procedures for distributional analysis: a unified framework based on the Shapley value. mimeo, University of Essex; 1999.

[38] Singh A. Inequality of opportunity in Indian children: the case of immunization and nutrition. Popul Res Policy Rev. 2011;30(6):861–83.

[39] Singh A. Inequality of opportunity in earnings and consumption expenditure: the case of Indian men. Rev Income Wealth. 2012;58(1):79–106.

[40] Soremekun O, Oyeyinka O, Haughton D. Sustainable development and inequality of opportunity in Africa. Sustainable industrialization in Africa. London: Palgrave Macmillan; 2016. pp. 173–99.

[41] Srivastava A, Mahmood SE, Mishra P, Shrotriya VP. Correlates of maternal health care utilization in Rohilkhand region, India. Annals Med Health Sci Res. 2014;4(3):417–25. 10.4103/2141-9248.133471 PMC4071744 24971219

[42] Story WT, Burgard SA, Lori JR. Tale of two countries: maternal health in Ghana and the united States. J Comp Eff Res. 2013;2(5):497–505.24236746

[43] Tsega Y, Endawkie A, Tsega G, Mekonen AM, Dawed YA, Stecher C. Trends and socioeconomic inequalities of recommended antenatal care services utilization in Ethiopia: A decomposition analysis using Ethiopian nationwide demographic health surveys 2011–2019. PL OS ONE. 2025; 20(2):e0318337. 10.1371/journal.pone.0318337. 10.1371/journal.pone.0318337 PMC11793738 39903763

[44] Wagstaff A. Poverty and health sector inequalities. Bull World Health Organ. 2002;80:97–105. 11953787 PMC2567730

[45] World Bank. World development report 2006: Equity and development. The World Bank; 2006.

[46] World Health Organization. World health statistics 2018: Monitoring health for the SDGs sustainable development goals. World Health Organization; 2018.

[47] World Health Organization. WHO health workforce support and safeguards list 2023. World Health Organization; 2023. 10.2471/BLT.23.290191 PMC10225944 37265683

[48] Youmbi AD, Nono BF, Akono CZ. Inequality in opportunity of access to antenatal care in cameroon: multilevel modelling, Spatial analysis and decomposition methods. Swiss J Econ Stat. 2024;160(1):5.

[49] Zakar R, Zakar MZ, Aqil N, Chaudhry A, Nasrullah M. Determinants of maternal health care services utilization in pakistan: evidence from Pakistan demographic and health survey, 2012–13. J Obstet Gynaecol. 2017;37(3):330–7. 27981860 10.1080/01443615.2016.1250728

