# Correction: Assessing inequality of opportunity in access to maternal healthcare services in Pakistan: A quantitative attempt

**DOI:** 10.1186/s12913-025-13525-8

**Published:** 2025-10-01

**Authors:** Jawad Rahim Afridi, Sajjad Ahmad Jan, Muhammad Farhan Asif

**Affiliations:** 1 Department of Economics, University of Peshawar, Peshawar, Pakistan; 2 UKM-Graduate School of Business, Universiti Kebangsaan Malaysia, Bangi, Selangor Malaysia; 3 Department of Business Management, Ilma University, Karachi, Pakistan

**Correction to: *BMC Health Services Research*** **(2025) 25:1167**

10.1186/s12913-025-13312-5

In this article, the author name Muhammad Farhan Asif was incorrectly written as Muhamad Farhan Asif. The author group has been updated above and the original article has been corrected.

